# The Shield of Self-Esteem: Buffering against the Impact of Traumatic Experiences, Fear, Anxiety, and Depression

**DOI:** 10.3390/bs14100901

**Published:** 2024-10-04

**Authors:** Alessandro Alberto Rossi, Silvia Francesca Maria Pizzoli, Isabel Fernandez, Roberta Invernizzi, Anna Panzeri, Federica Taccini, Stefania Mannarini

**Affiliations:** 1 Department of Philosophy, Sociology, Education, and Applied Psychology, Section of Applied Psychology, University of Padova, 35131 Padova, Italy; federica.taccini@unipd.it (F.T.); stefania.mannarini@unipd.it (S.M.); 2 Center for Intervention and Research on Family Studies—CIRF, Department of Philosophy, Sociology, Education, and Applied Psychology, Section of Applied Psychology, University of Padova, 35131 Padova, Italy; 3 Humane Technology Laboratory, Catholic University of the Sacred Heart, 20123 Milan, Italy; silviafrancesca.pizzoli@unicatt.it; 4 Department of Psychology, Catholic University of the Sacred Heart, 20123 Milan, Italy; 5 Associazione EMDR Italia, 20123 Milan, Italy; isabelf@emdritalia.it; 6 Child Neurology and Psychiatry Unit, ASST Lecco, 23900 Lecco, Italy; ro.invernizzi@asst-lecco.it; 7 Department of Medicine and Surgery, University of Milano-Bicocca, 20126 Milan, Italy; 8 Department of General Psychology, University of Padova, 35131 Padova, Italy; anna.panzeri@unipd.it

**Keywords:** trauma, post-traumatic stress, PTSD, PTSS, anxiety, depression

## Abstract

Background: Adverse life occurrences (e.g., severe accidents, violence/abuse, organic disorders such as COVID-19) can elicit traumatic responses that heighten fear, anxiety, and depression. However, scientific research has shown that certain variables, such as self-esteem, based on theories like terror management theory (TMT) and the anxiety-buffering hypothesis (ABH), can mitigate the negative effects of trauma. This study aimed to test the ABH by assessing the buffering role of self-esteem in the relationships among the impact of traumatic experiences, fear, anxiety, and depression. Method: An observational research design was used. This study involved 321 participants who experienced COVID-19 as a traumatic experience. A sequential multiple-mediation model with observed variables (path analysis) was used to test the impact of the traumatic experience on fear, anxiety, and depression, examining the protective role of self-esteem. Results: A path analysis revealed that fear and anxiety mediated the relationship between the impact of the traumatic experience of COVID-19 and depression. Additionally, in line with the ABH, self-esteem was found to mediate the relationship between the predictors and their adverse psychological consequences. This suggests that self-esteem played a buffering role, mitigating the negative impact of traumatic experiences on mental health outcomes. Conclusions: These findings underscore the central mediating role of self-esteem, as well as fear and anxiety, in the pathway from trauma-related factors to depression. These insights advocate for evidence-based interventions aimed at alleviating the psychological suffering associated with traumatic experiences, fostering adaptation, and supporting psychological health.

## 1. Introduction

Traumatic experiences can deeply affect individuals, causing considerable distress and disruption in their lives [1,2]. This impact can arise from both overt, life-threatening scenarios and more subtle types of trauma [3,4,5]. No matter the specific nature of the traumatic event, the psychological effects can be significant and enduring, often interfering with a person’s overall ability to function effectively [6,7].

Life-threatening situations that can have varying degrees of traumatic impact include wars, natural disasters, severe injuries, and illnesses such as cancer, stroke, or COVID-19 [8,9,10]. The COVID-19 pandemic posed a significant threat to global health due to the lack of prior immunity and effective treatment options [11,12]. In the initial phases of the pandemic, the virus caused a wide range of symptoms, from asymptomatic cases to severe complications such as acute respiratory distress syndrome and death [13,14]—especially in older adults and individuals with underlying medical conditions [15,16,17,18].

Moreover, some recent evidence from scientific literature suggested that the COVID-19 pandemic can represent a case of collective trauma, as it was impacting all the individuals of the society at the same time, sharing the same restrictions and the same fears [19,20]. Indeed, considering the severity of some COVID-19 infections and the pandemic itself as a significant traumatic event, the COVID-19 outbreak has severely impacted mental health, increasing the prevalence and severity of post-traumatic stress symptoms (PTSS) and post-traumatic stress disorder (PTSD) symptoms across various populations. Exposure to traumatic experiences is characterized by three key symptom clusters: intrusive re-experiencing of the traumatic event, avoidance behaviors, and hyperarousal [3,7,21,22]. These symptoms can significantly disrupt an individual’s daily functioning and overall quality of life, underscoring the importance of timely and effective interventions to address the psychological consequences of trauma [23,24,25].

The impact of traumatic events can have a profoundly negative impact on an individual’s mental health and well-being. A traumatic event refers to a specific major incident that causes harm or threat, while a traumatic experience is the subjective emotional response and psychological impact that follows an event that also may not be necessarily considered ‘major’ in itself but is subjectively evaluated as such [26]. Thus, focusing on traumatic experiences instead of traumatic events allows for including a broader variety of circumstances which could lead to PTSS and PTSD, beyond the most evident ones.

Traumatic experiences represent a significant risk factor for the development and persistence of PTSS, which can manifest in a range of psychological difficulties [2,27,28,29]. These difficulties may include PTSD, fear, anxiety, and depression [7,30,31]. Moreover, the magnitude of the pandemic had a severe psychological impact, causing substantial mental health issues [32,33,34,35,36].

In particular, the fear of COVID-19 contributed to the development of anxiety, characterized by persistent worry, negative expectations, and concern for one’s own or others’ well-being [37,38,39,40,41,42]. Furthermore, according to several studies, anxiety may further trigger depressive symptoms, including sadness, negative self-perception, and loss of positive emotions [41,43,44,45]. Depressive symptoms have become widespread during the pandemic and were linked to an increased risk of anticonservative behaviors [46].

In this context, the Terror Management Theory (TMT) posits that individuals’ awareness of their own mortality, which can be heightened by experiencing traumatic events such as accidents, violence, or severe illness, generates profound fears of death [47]. This awareness of mortality conflicts with the innate human desire to live and survive, leading to increased anxiety and depression [6,41,47].

However, research over the past two decades has identified certain psychological factors that can serve as a protective shield against the negative consequences of traumatic experiences, such as anxiety and depression [48]. The anxiety-buffering hypothesis (ABH), derived from TMT, suggests that self-esteem plays a crucial role in buffering the relationship between the impact of traumatic experiences and the development of anxiety and depressive symptoms [6,41,48,49]. Self-esteem is conceptualized as the beliefs, evaluations, and attitudes that individuals hold towards themselves, which are rooted in their personal values and the cultural and social context in which they are embedded [50]. By meeting the standards and expectations of their cultural worldview, individuals can gain social validation and a sense of personal value and meaningful role in society [51,52]. According to the ABH, this reconnection to a broader universe of personal meanings and values through self-esteem can serve as a protective buffer against the detrimental psychological effects of life-threatening stressors, traumatic events, and traumatic experiences [49]. Consequently, self-esteem is hypothesized to play a crucial role in mitigating the negative psychological consequences, such as anxiety and depression, that often arise in the aftermath of traumatic experiences.

About the rationale of the present study, it is supported by the large amount of literature regarding the interplay of self-esteem, post-traumatic symptoms, fear, anxiety, and depression applied to the context of the COVID-19 pandemic. However, few studies tested the circumstances in which the ABH holds or not, also examining the relationships among constructs and their nature.

Building on the theoretical background, this study was aimed to test the ABH within the context of the traumatic impact of the COVID-19 pandemic. Specifically, the study examined whether self-esteem could serve as a protective factor, buffering the relationships between the impact of traumatic experiences, fear of COVID-19, and the development of anxiety and depression.

The following research hypotheses (RH) were formulated:

RH#1: post-traumatic stress symptoms, fear of COVID-19, anxiety symptoms, and depression symptoms are all positively related to each other; at the same time, self-esteem should be negatively correlated with the abovementioned constructs;

RH#2: post-traumatic stress symptoms will be positively associated with depression symptoms through the positive mediating role of fear of COVID-19 and anxiety symptoms. However, these relationships will be buffered (negatively associated) by the effect of self-esteem.

In other words, it was hypothesized that the relationships of the post-traumatic stress symptoms and depression symptoms should be mediated by fear of COVID-19 and anxiety symptoms, but self-esteem would play a buffering role, mitigating the negative psychological impact of these pandemic-related factors.

## 2. Methods

### 2.1. Study Desing

The primary elements of the design were focused on examining the relationships between post-traumatic symptoms, fear of COVID-19, self-esteem, anxiety, and depression. An observational cross-sectional study was conducted, utilizing data collected through an online self-report survey. A snowball sampling method [53] was used to recruit participants from the general population via social media platforms.

### 2.2. Setting and Procedure

This study’s setting was entirely online, with participants recruited via social media platforms. Data were collected in 2021 when the worst of the pandemic was over but there were still several COVID-19 cases requiring partial lockdown restrictions. This study was approved by the Ethics Committee of the University of Padua (protocol no. 3565).

### 2.3. Inclusion Criteria

The inclusion criteria were as follows: (A) having experienced COVID-19 as a traumatic experience with a clear remembrance of it; (B) providing complete responses; (C) being a native Italian speaker; (D) being 18 years or older; and (E) providing written informed consent.

### 2.4. Sample Size Determination

According to the study design, the sample size was planned a priori using a simulation study according to the procedure described by Wang and Rhemtulla (2021) [54] and setting the desired effects following the guidelines of Cohen (1988) [55]. Specifically, the regression coefficients were set to have a minimal association among them (effect size; standardize regression coefficient = 0.25). The Type I error (alpha) was set at 0.050. The minimum desired power (1−β) for the regression coefficients was chosen to be equal to 0.80 [55]. The number of simulations was 1000. As suggested, different sample sizes were simulated to identify the minimum number of subjects that would ensure a minimum power equal to (or greater than) the established one for all the model coefficients. Consequently, the simulation analysis reported a minimum number of subjects equal to 293.

### 2.5. Measures

The socio-demographic information collected included sex, age, education, employment status, etc. Table 1 presents the sample characteristics.

In the initial part of the survey, the materials outlining the aims and objectives of the research explicitly required that participants must have experienced COVID-19 as a traumatic event. Additionally, to assess the eligibility of participants, they were asked again whether they experienced COVID-19 as a traumatic event, through a single dichotomous (yes/no) item. Additionally, the following self-report measures were administered.

#### 2.5.1. Post-Traumatic Symptom Questionnaire (PTSQ)

The PTSQ [6] is an accurate, robust, and reliable questionnaire consisting of two separate sections [7]—which can be used together or independently of each other—designed to evaluate the following: (1) the presence of traumatic events and (2) PTSS and its three core domains related to the impact of traumatic experiences: (A) intrusivity, (B) avoidance, and (C) hyperarousal. The first section includes a checklist (PTSQ-CL: 0 = No; 1 = Yes) that evaluates the presence of traumatic events during both childhood/adolescence and adulthood, with higher scores indicating a greater number of traumatic events. Furthermore, in accordance with the study’s inclusion criteria, this first section was not administered. The second section features 12 items that assess post-traumatic symptoms across the three core trauma components and provides a total score (PTS). Participants rate each symptom on a 5-point Likert-type scale (1 = “not at all” to 5 = “extremely”), with higher scores reflecting greater severity in each domain. A total score (post-traumatic symptoms: PTS) assesses the overall severity of PTSS.

For the present study, a short 6-item version was used (i.e., PTSQ-SF), the psychometric properties of which have been extensively investigated and are reported in the Supplementary Materials. In this study, the PTSQ-SF reported good internal consistency values: McDonald’s omega = 0.815.

#### 2.5.2. Fear of COVID-19 (FCV-19S)

The FCV-19S [56,57] is a 7-item self-report scale designed to evaluate cognitive, emotional, behavioral, and physiological manifestations of fear related to COVID-19. Participants rate their agreement with each statement on a 5-point Likert-type scale (1 = “strongly disagree” to 5 = “strongly agree”), with higher scores indicating greater fear of COVID-19. In this study, the FCV-19S demonstrated good internal consistency: McDonald’s Omega = 0.879.

#### 2.5.3. Self-Esteem

The RSE [58,59] is among the most widely utilized self-report measures for assessing overall self-esteem across clinical and general populations. It consists of 10 statements evaluating individuals’ feelings about themselves. Participants indicate their level of agreement with each statement on a 4-point Likert-type scale (1 = “not at all” to 4 = “always”), with higher scores indicating greater global self-esteem. In this study, the RSE demonstrated good internal consistency: McDonald’s Omega = 0.808.

#### 2.5.4. Anxiety Symptoms (ANX)

The anxiety scale of the Brief Symptom Check-List [60] is a 10-item self-report tool that assesses several symptoms of anxiety. Participants rate the severity of their symptoms on a 5-point Likert-type scale (1 = “not at all” to 5 = “always”), with higher scores suggesting more severe anxiety symptoms. In this study, the ANX subscale demonstrated high internal consistency: McDonald’s Omega = 0.934.

#### 2.5.5. Depression Symptoms (DEP)

The depression scale of the Brief Symptom Check-List [60] is a 13-item self-report tool that assesses several manifestations of depression. Participants rate the severity of their symptoms on a 5-point Likert-type scale (1 = “not at all” to 5 = “always”), with higher scores suggesting more severe depressive symptoms. In this study, the DEP subscale demonstrated good internal consistency: McDonald’s Omega = 0.911.

### 2.6. Statistical Analysis

The statistical analyses were performed using the R software and the following packages: lavaan [61], psych [62], semPlot [63], and tidyverse [64].

To attain the objective of this study, several phases were run. *First*, before carrying out the main analyses of the study, preliminary analyses were performed to assess the intensity of the relationships between variables [65] using Pearson’s correlation coefficient and interpreted using Cohen’s benchmarks [55]: *r* from 0.10 to 0.30, small; *r* from 0.30 to 0.50, moderate; *r* > 0.50, large. Also, model assumptions were tested, and no violations were detected [65,66].

**Figure 1:**
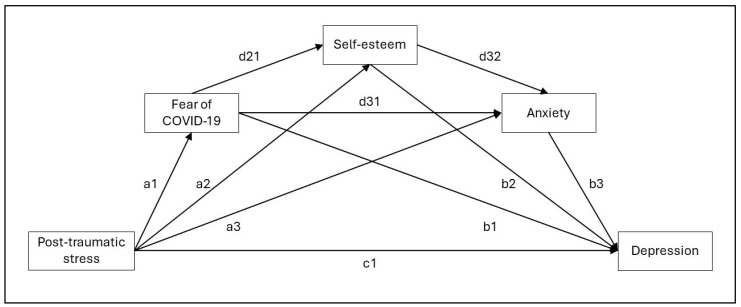
Sequential multiple-mediation model—conceptual representation.

*Second*, a sequential multiple-mediation model with observed variables (i.e., path analysis) was performed [67,68,69,70,71]. In particular, the PTSS related to the impact of traumatic event (PTSQ-SF; namely, X) was regressed on fear of COVID-19 (FCV-19S; M1), self-esteem (RSE; M2), anxiety (ANX; M3), and depression (DEP; Y) (see Figure 1).

*Third*, to carry out the multiple-mediation model, the maximum likelihood (ML) estimator was used with a 10,000 bootstrap resampling procedure with the Bollen–Stine method to deal with non-perfect normality of variables. Furthermore, it is important to emphasize that classic fit indices (i.e., RMSEA, CFI, SRMR [66,72]) are unnecessary because a saturated model with observed variables was specified—thus exhibiting perfect-fit indices [67,68]. For this reason, reporting them is redundant [67,73]. All regression coefficients reported in the results section were unstandardized (β).

## 3. Results

### 3.1. Participants

A sample of 321 participants was enrolled, all of whom stated experiencing COVID-19 as a traumatic experience. In accordance with the inclusion/exclusion criteria, there were no missing data. The sample consisted of 65 males (20.2%) and 256 females (79.8%), aged between 18 and 78 years (mean = 39.60, SD = 12.44). Further details are provided in Table 1.

### 3.2. Preliminary Analysis

The correlation analyses indicated that the psychological variables in the sequential multiple-mediation model were related to one another, with different strengths—ranging from small to large in magnitude (Table 2).

### 3.3. Sequential Multiple-Mediation Model

**Figure 2:**
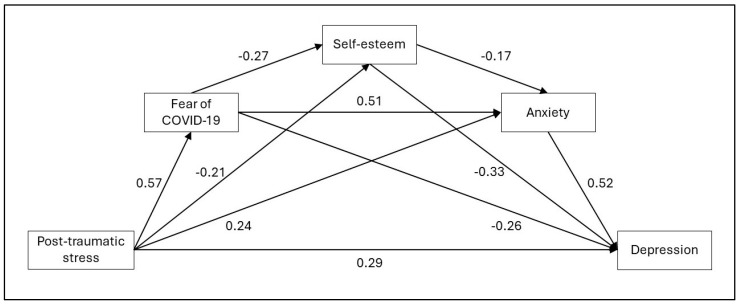
Results of the sequential multiple-mediation model (standardized regression coefficients).

The sequential multiple-mediation mode model (Figure 1 and Figure 2) provided hypothesized results (see Table 3).

The impact of traumatic events—PTSQ-SF (PTSS; X)—was positively associated with fear of COVID-19 (FCV-19S; M1), *path a11*: β = 0.661 (SE = 0.051) [95%CI: 0.560; 0.761], *z* = 13.026, *p* < 0.001. According to the ABH, fear of COVID-19 (M1) was negatively associated with self-esteem (RSE; M2), *path d21*: β = −0.186 (SE = 0.043) [95%CI: −0.271; −0.103], *z* = −4.357, *p* < 0.001. Also, self-esteem (M2) was negatively associated with anxiety (ANX; M3), *path d31*: β = −0.035 (SE = 0.008) [95%CI: −0.050; −0.020], *z* = −4.559, *p* < 0.001—thus highlighting the buffering effect of self-esteem. Lastly, anxiety (M3) was positively associated with depression (DEP; Y), *path b3*: β = 0.476 (SE = 0.051) [95%CI: 0.375; 0.576], *z* = 9.344, *p* < 0.001.

In addition, in line with the ABH, self-esteem (M2) was negatively associated with both the impact of traumatic event (X→ M2; *path a2*: β = −0.169 (SE = 0.048) [95%CI: −0.262; −0.074], *z* = −3.534, *p* < 0.001) and depression (M2 → Y; *path b2*: β = −0.065 (SE = 0.008) [95%CI: −0.080; −0.049], *z* = −8.290, *p* < 0.001).

Moreover, in line with the hypothesis, the impact of a traumatic event was positively associated with both anxiety (X→ M3; *path a3*: β = 0.041 (SE = 0.007) [95%CI: 0.028; 0.056, *z* = 5.787, *p* < 0.001) and depression (X → Y; *path c1*: β = 0.045 (SE = 0.007) [95%CI: 0.031; 0.059], *z* = 6.167, *p* < 0.001). Lastly, fear of COVID-19 (M1) and anxiety (M3) were positively associated; *path d31*: β = 0.075 (SE = 0.007) [95%CI: 0.062; 0.088], *z* = 11.178, *p* < 0.001).

The total indirect effect (impact of traumatic experience → fear of COVID-19 → self-esteem → anxiety → depression) was statistically significant: β = 0.002 (SE = 0.001) [95%CI: 0.001; 0.004], *z* = 2.789, *p* = 0.005. Moreover, the total model effect was also statistically significant: β = 0.089 (SE = 0.007) [95%CI: 0.076; 0.102], *z* = 13.315, *p* < 0.001]—revealing a partially mediated model that highlighted the buffering effect of self-esteem. Table 3 reports all direct and indirect effects calculated for the present model. In addition, the degree of explained variance was 58.7% (*R*^2^ = 0.587).

## 4. Discussion

According to the ABH, the present study aimed at testing the buffering effect of self-esteem on the relationship between the impact of traumatic experience, fear, anxiety, and depression.

Findings from this research shed light on the buffering role of self-esteem on the relationships among the impact of traumatic experiences, fear, anxiety and depression, using a sequential multiple-mediation model. The trauma-related variables (i.e., impact of traumatic experiences, fear, anxiety, and depression) were reciprocally correlated with moderate-to-high positive and statistically significant positive associations (RH#1), proving their interrelation in structuring the psychological reactions to traumatic experiences [74]. Additionally, as hypothesized (RH#1), self-esteem revealed small–moderate negative and statistically significant association with all of the variables related to the impact of traumatic experiences.

In line with the scientific literature, this study has shown that the impact of a traumatic experience—such as the COVID-19 pandemic—is associated with anxiety and depression. Indeed, the multiple-mediation model shows a positive relationship between the impact of the traumatic experience, anxiety, and depression, indicating that as the impact of the traumatic experience increases, anxiety and depression also increase. Specifically, controlling for all other variables in the model, an increase of 1 point in the impact of the traumatic experience was associated with an increase of 0.041 points (unstandardized coefficient) in anxiety and 0.045 points (unstandardized coefficient) in depression. These results once again confirm the powerful effect of traumatic experiences on individuals’ mental health, identifying it as a major risk factor for common psychopathological disorders.

Additionally, the fear of COVID-19 is also associated with anxiety and depression [75], further confirming that prolonged states of fear can lead to anxiety and depression [41,76,77,78]. Specifically, controlling for the impact of the traumatic experiences, the model showed that an increase of 1 point in the fear of COVID-19 resulted in an increase of 0.075 points (unstandardized coefficient) in anxiety and −0.035 points (unstandardized coefficient) in depression. It should be noted that when controlling for anxiety activation, the data revealed a negative association between fear and depression (−0.035). This finding aligns with previous research [41] and can be attributed to the distinct nature of these emotional states. Fear represents an activating emotion that prompts a “fight or flight” response, whereas depression is characterized by a generalized deactivation, manifested in slowed behavior, impaired cognition, and flattened affect [32,79]. Importantly, in line with previous studies [41], fear was positively and strongly associated with anxiety [37,38], which may then lead to depression [76]—suggesting a partially mediated model, where fear first leads to anxiety, which in turn contributes to the development of depressive symptoms. In line with the literature, these results suggest that a prolonged state of fear may lead to the development of adverse psychological symptoms, representing an important risk factor for the onset of psychopathological symptoms.

However, in perfect alignment with the hypotheses and previous literature, the sequential multiple-mediation model highlighted the buffering role of self-esteem (RH#2). Specifically, self-esteem mitigated the pathways leading from the impact of the traumatic experience to depression symptoms, through states of fear and anxiety. In line with the ABH and TMT [47,49], due to its buffering effect, self-esteem showed negative relationships with all trauma-related psychological variables (all β values were negative), hindering the total effects of these relationships. However, while the results are encouraging, it is important to note that this is a partial mediation model, as all relationships between trauma-related variables maintain an effect despite the buffering role of self-esteem. Additionally, it is worth noting that although self-esteem plays a crucial role in protecting individuals from these negative variables, its effects are relatively modest—in line with recent studies [6,41]. An increase of 1 point in self-esteem was associated with a decrease of −0.035 points (unstandardized coefficient) in anxiety and −0.065 (unstandardized coefficient) points in depression, suggesting the need to identify variables that act as buffers but with a greater effect in mitigating adverse effects.

Moreover, it should be highlighted that, in line with previous studies and considering both theoretical and statistical reasons, a mediation model was preferred over a moderation model. Theoretically, a mediation approach is closer and more correlated to the original ABH and TMT frameworks [47,49], which conceptualize self-esteem as an intermediate buffer between life-threatening stressors and anxiety [51]. Indeed, self-esteem not only influences individuals’ levels of anxiety and depression but is also influenced by negative psychological states, such as the impact of traumatic experiences, fear, and loneliness, triggering negative cognitions and emotions that significantly impact the idea of oneself [47,80]. Research shows that a traumatic experience as well as fear can threaten self-evaluation [47]. More specifically, the impact of negative experiences can activate negative cognitions and emotions that significantly influence self-concept (e.g., “I am a failure”, “I am worthless”) [79,81], thus leaving scars on self-concept and persistently threatening and reducing self-esteem and self-efficacy [80]. Therefore, a moderation approach would not be suitable, given the theoretical background of this study, and would not adequately account for the complexity of relationships among the psychological constructs considered.

Given the impact of traumatic experiences on mental health, it is crucial to assess it in both clinical settings to provide prompt psychological interventions and in research to account for its effects on other constructs. The scientific literature has highlighted the significant negative traumatic impact and adverse psychological consequences that the COVID-19 pandemic has had on individuals worldwide [13,82,83]. This traumatic impact of the COVID-19 pandemic has generated intense fear and anxiety about infection, illness, and thoughts of death in the population, which have converged into feelings of depression [84,85,86]. Therefore, both the traumatic impact of the disease and the fear of COVID-19 have been significant risk factors for the development of anxiety and subsequent depression [87,88,89,90].

In summary, the results showed that self-esteem acts as a shield protecting against fear and anxiety triggered by the impact of the traumatic experience. Therefore, these findings confirmed the validity of the ABH in the context of the COVID-19 pandemic. The results also highlighted that both the impact of the traumatic experience and fear can trigger unbearable feelings of anxiety, which in turn are strongly linked to depression.

### 4.1. Limitations

This research is not free of limitations. The study relied on self-assessment tools, which may have introduced an element of response bias. Additionally, no clinical interviews were conducted to ascertain the impact of trauma or to formally diagnose PTSD. However, it is important to note that all self-report questionnaires used are well-validated and have robust psychometric properties, providing consistent results in measuring specific psychological constructs, thereby reducing potential measurement bias. In this cross-sectional research design, causal relationships cannot be demonstrated, but reliable statistical methodologies allowed testing hypotheses regarding associations and direct influences among constructs. Future research could employ longitudinal designs to investigate the impact of trauma over time [91,92,93,94]. This study considered a limited number of variables, and potential confounding variables (such as anxiety sensitivity, difficulties in emotion regulation, social support) may have an effect, which could be examined in future studies [95,96,97]. Lastly, recruitment through social media helped reduce selection bias by enabling access to a diverse range of participants. However, the snowball sampling method may have introduced bias due to the overrepresentation of certain social groups, as acknowledged in the study’s limitations. Additionally, despite efforts to ensure a representative sample, the disproportionate number of females (79.8%) may have introduced gender-related bias in the findings. Future studies should consider accounting for both gender and ethnic differences [98].

### 4.2. Strengths

Despite its limitations, this study has several notable strengths that contribute to its overall value. First, it is grounded in a robust theoretical framework, supported by an extensive body of experimental and longitudinal research [49,51,99], which lends credibility to the study’s hypotheses and findings. This theoretical foundation ensures that the research is not conducted in isolation but builds upon a well-established understanding of trauma and psychological responses. Second, the study utilized a carefully selected and sufficiently large sample, which was determined using a simulation-based approach to ensure adequate statistical power. The inclusion of participants across different age groups who had experienced COVID-19 as a traumatic event adds to the generalizability of the findings, allowing for a broader understanding of how different demographics may respond to such a global crisis. Third, the research employed reliable and validated self-report measures, coupled with advanced statistical techniques that are widely recognized in psychological research. The use of path analysis and multiple-mediation models ensures that the complex relationships between variables, such as trauma, fear, anxiety, depression, and self-esteem, are adequately captured and analyzed. This methodological precision strengthens the reliability and validity of the study’s conclusions. Importantly, this study not only corroborates the results of previous research but also extends them in significant ways. By exploring the intricate relationships between the impact of traumatic experiences, fear of COVID-19, anxiety, and depression, the study offers new insights into the psychological effects of the pandemic. Additionally, it highlights the protective role of self-esteem, suggesting that higher levels of self-esteem may buffer individuals from the more severe psychological consequences of trauma. This finding opens new avenues for interventions aimed at supporting self-esteem as a potential means of mitigating the impact of traumatic events.

### 4.3. Implications for Clinical and Research Practice

The implications of the present research are relevant both for the research and clinical practice. Regarding the clinical implications of this study, its results suggest a possible intervention strategy to provide psychological support to people suffering from the emotional consequences of traumatic experiences, such as fear, anxiety, and depression, in order to alleviate the onset of psychological difficulties. According to the ABH, if self-esteem provides protection against stressors, such stressors should increase the need for self-esteem to alleviate psychological burden [100]. Consequently, enhancing self-esteem should act as a buffer against anxiety, reducing negative psychological issues in response to threats or stressors. Therefore, among various possible interventions, psychological interventions aimed at increasing (directly or indirectly) self-esteem may represent an effective strategy to mitigate distressing psychological responses to the impact of the traumatic experience and the resulting fear, especially among vulnerable populations such as individuals with psychiatric disorders or those at risk of domestic violence [5,101].

In this regard, an integrated psychological intervention strategy that also addresses the direct impact of the traumatic experience could lead to greater benefits for the patient. Meta-analytic evidence has demonstrated the effectiveness of specific psychotherapeutic treatments in reducing symptoms of PTSS as well as PTSD [102]. Among these treatments, Eye Movement Desensitization and Reprocessing (EMDR) therapy [103] has been identified as one of the treatments recommended by the World Health Organization [104]. A recent systematic review has shown that in all considered studies, EMDR improved PTSD symptoms in hospitalized adult patients [105]. Scientific evidence has indicated that the mechanisms of action of EMDR are based on modifications of neuroanatomical pathways [106] and the recoding of aversive traumatic memories [107]. While these results are not definitive, they support the use of evidence-based treatments for PTSD.

## 5. Conclusions

Summarizing, this study provides additional support for the protective role of self-esteem against fear, anxiety, and depression related to the impact of traumatic experiences—highlighting that the ABH as well as the TMT are well-founded theoretical framework that offer clinically relevant insights, even in contexts related to organic illnesses.

These findings can also be valuable in helping clinicians develop effective and personalized interventions to enhance individuals’ mental health, with particular attention to more vulnerable populations. Specifically, psychological interventions aimed at supporting and enhancing the protective role of self-esteem should be conceptualized to adequately support individuals suffering from the impact of traumatic experiences—perhaps integrating with more established psychotherapy protocols (e.g., EMDR)—in order to minimize the psychological burden of illness while promoting adaptation and positive psychological health outcomes. In conclusion, in a broader sense, these results could be extended to alleviate the psychological burden of dysfunctional psychological reactions in response to physical and/or psychological illnesses.

## Figures and Tables

**Table 1:** Samples’s descriptive statistics.

	(*N* = 321)	
Age (*M*. *SD*)	39.60	12.44
Sex (*n*. %)		
Male	65	20.2%
Female	256	79.8%
Marital status (*n*. %)		
Single	67	20.9%
In a relationship	96	29.9%
Married	134	41.7%
Separated/divorced	20	6.2%
Widowed	4	1.2%
Education (*n*. %)		
Middle school	40	12.4%
High school	130	40.5%
University	123	38.3%
Ph.D.	28	8.7%
Work status (*n*. %)		
Student	30	9.3%
Full-time worker	177	55.1%
Entrepreneur	57	17.8%
Housewife	14	4.4%
Unemployed	22	6.9%
Retired	21	6.5%

**Table 2:** Correlations among measures.

		Descriptive Statistics				Correlations				
		M	SD	Sk	K	1	2	3	4	5
1	PTS	17.807	4.678	−0.134	−0.069	-				
2	Fear of COVID-19	19.701	5.430	0.248	−0.447	0.569	-			
3	Self-Esteem	29.274	3.762	0.008	0.695	−0.363	−0.388	-		
4	Anxiety	1.020	0.802	0.813	−0.207	0.589	0.708	−0.450	-	
5	Depression	1.158	0.733	0.628	−0.225	0.565	0.402	−0.569	0.655	-

Note: all correlations are statistically significant with *p* < 0.001. M = mean; SD = standard deviation; Sk = skewness; K = Kurtosis. PTS = PTSQ-SF; Fear of COVID-19 = FCV-19S; Self-Esteem = RSE; Anxiety = ANX subscale of the BSCL; Depression = DEP subscale of the BSCL.

**Table 3:** Results of the sequential multiple-mediation model.

Path		β*	β (SE)	95%CI [L–U]	*z*-Value	*R* ^2^
PTSS (X) → Fear of COVID-19 (M1)	(a1)	0.569	0.661 (0.051)	[0.560; 0.761]	13.026	0.324
Fear of COVID-19 (M1) → Self-Esteem (M2)	(d21)	−0.269	−0.186 (0.043)	[−0.271; −0.103]	−4.357	0.180
Self-Esteem (M2) → Anxiety (M3)	(d32)	−0.166	−0.035 (0.008)	[−0.050; −0.020]	−4.559	0.575
Anxiety (M3) → Depression (Y)	(b3)	0.521	0.476 (0.051)	[0.375; 0.576]	9.344	0.587
PTSS (X) → Self-Esteem (M2)	(a2)	−0.210	−0.169 (0.048)	[−0.262; −0.074]	−3.534	
PTSS (X) → Anxiety (M3)	(a3)	0.239	0.041 (0.007)	[0.028; 0.056]	5.787	
Fear of COVID-19 (M1) → Anxiety (M3)	(d31)	0.508	0.075 (0.007)	[0.062; 0.088]	11.178	
Fear of COVID-19 (M1) → Depression (Y)	(b1)	−0.258	−0.035 (0.007)	[−0.048; −0.021]	−4.918	
Self-Esteem (M2) → Depression (Y)	(b2)	−0.332	−0.065 (0.008)	[−0.080; −0.049]	−8.290	
PTSS (X) → Depression (Y)	(c1)	0.285	0.045 (0.007)	[0.031; 0.059]	6.167	
Indirect effect of X on Y via M1	(a1*b1)	−0.147	−0.023 (0.005)	[−0.033; −0.014]	−4.705	
Indirect effect of X on Y via M2	(a2*b2)	0.070	0.011 (0.003)	[0.005; 0.018]	3.214	
Indirect effect of X on Y via M3	(a3*b3)	0.125	0.020 (0.004)	[0.013; 0.028]	5.121	
Indirect effect of X on Y via M1 and M2	(a1*d21*b2)	0.051	0.008 (0.002)	[0.004; 0.013]	3.688	
Indirect effect of X on Y via M1 and M3	(a1*d31*b3)	0.151	0.024 (0.004)	[0.017; 0.032]	6.185	
Indirect effect of X on Y via M2 and M3	(a2*d32*b3)	0.018	0.003 (0.001)	[0.001; 0.005]	2.778 ^	
Indirect effect of X1 on Y via M1, M2, and M3	(a1*d21*d32*b3)	0.013	0.002 (0.001)	[0.001; 0.004]	2.789 ^	
Total effect		0.565	0.089 (0.007)	[0.076; 0.102]	13.315	

Note: All coefficients are statistically significant with *p* < 0.001, except for: ^ *p* = 0.005; β* = standardized beta; β = unstandardized beta; 95%CI = 95% confidence intervals for the unstandardized beta; *R*^2^ = explained variance. PTSS = PTSQ-SF; Fear of COVID-19 = FCV-19S; Self-Esteem = RSE; Anxiety = ANX subscale of the BSCL; Depression = DEP subscale of the BSCL.

## Data Availability

The datasets presented in this article are not readily available because due to privacy restrictions, data were available from the corresponding author on a reasonable request.

## Supplementary Materials

The following supporting information can be downloaded at https://www.mdpi.com/article/10.3390/bs14100901/s1; File S1: The Italian and English versions of the PTSQ-SF and the validation analyses. References [108,109,110,111,112,113,114,115,116] are cited in the Supplementary Materials.
