# Supplementary material for: The Shield of Self-Esteem: Buffering against the Impact of Traumatic Experiences, Fear, Anxiety, and Depression
Source: Behav Sci (Basel). 2024 Oct 4;14(10):901. doi: 10.3390/bs14100901 (PMC11505037; doi:10.3390/bs14100901)
Supplement: Supplementary file 1 [file behavsci-14-00901-s001.zip › behavsci-3108687-supplementary.pdf]

The Shield of Self-Esteem: Buffering Against the Impact of Traumatic Events, Fear, Anxiety, and Depression

Alessandro Alberto Rossi *, Silvia Francesca Maria Pizzoli, Isabel Fernandez, Roberta Invernizzi, Anna Panzeri, Federica Taccini, Stefania Mannarini

SUPPLEMENTARY MATERIAL

Psychometric properties of the Post-Traumatic Symptom Questionnaire – Short Form

(PTSQ-SF)

CORRESPONDING AUTHOR:

Alessandro Alberto Rossi

Department of Philosophy, Sociology, Education, and Applied Psychology, University of Padova, Padova, Italy

Email:a.rossi@unipd.it

Index

Structural validity 3

Results 4

PTSQ-SF – English version.. 6

PTSQ-SF – Italian version.. 7

References 8

Structural validity

Statistical Analysis

According to the guidelines, the measurement model of the PTSQ-SF was tested by means of confirmatory factor analysis (CFA) [72,108].

Using procedures widely available in the scientific literature [109,110], the questionnaire was reduced from 12 to 6 items – indeed, the original version of the PTSQ [111] included 4 items per dimension: thus, 4 items for the intrusion dimension, 4 items for the avoidance dimension, and 4 items for the hyper-arousal dimension. Specifically, compared to the original version of the PTSQ (12 items), the reduced version (PTSQ-SF) retained only two items per dimension – for a total of 6 items (see Figure S2).

For the analysis of the factorial structure, a first-order model with a single latent factor was specified, in which each of the six items was loaded onto that single latent factor. Additionally, considering the semantic content of the items, the residuals of these items were correlated with each other [72,111–115].

The diagonally weighted least square (DWLS) estimator was used as the response scale of each questionnaire should be considered as a categorical response variable [72,66].

Model fit was assessed with the conventional goodness-of-fit indices and their recommended cutoff values [66,72,116]: (A) the Chi-square statistics ($\chi 2$) that should preferably be non-statistically significant ($p > 0.05$); (B) the Root-Mean-Square Error of Approximation (RMSEA), with 'RMSEA < 0.08' indicating 'acceptable' model fit; (C) the Comparative Fit Index (CFI), with 'CFI > 0.90' indicating 'acceptable' model fit; and (D) the Standard Root Mean Square Residual (SRMR), with 'SRMR < 0.08' indicating good model fit.

Results

The PTSQ-SF provided good fit indices: S-Bχ^2 (6) = 10.302; $p < .001$; RMSEA = 0.047; 90%CI: 0.000-0.095; p(RMSEA < 0.05) = 0.472, CFI = 0.997, SRMR = 0.036. See Figure S1 and Table S1 for further details.

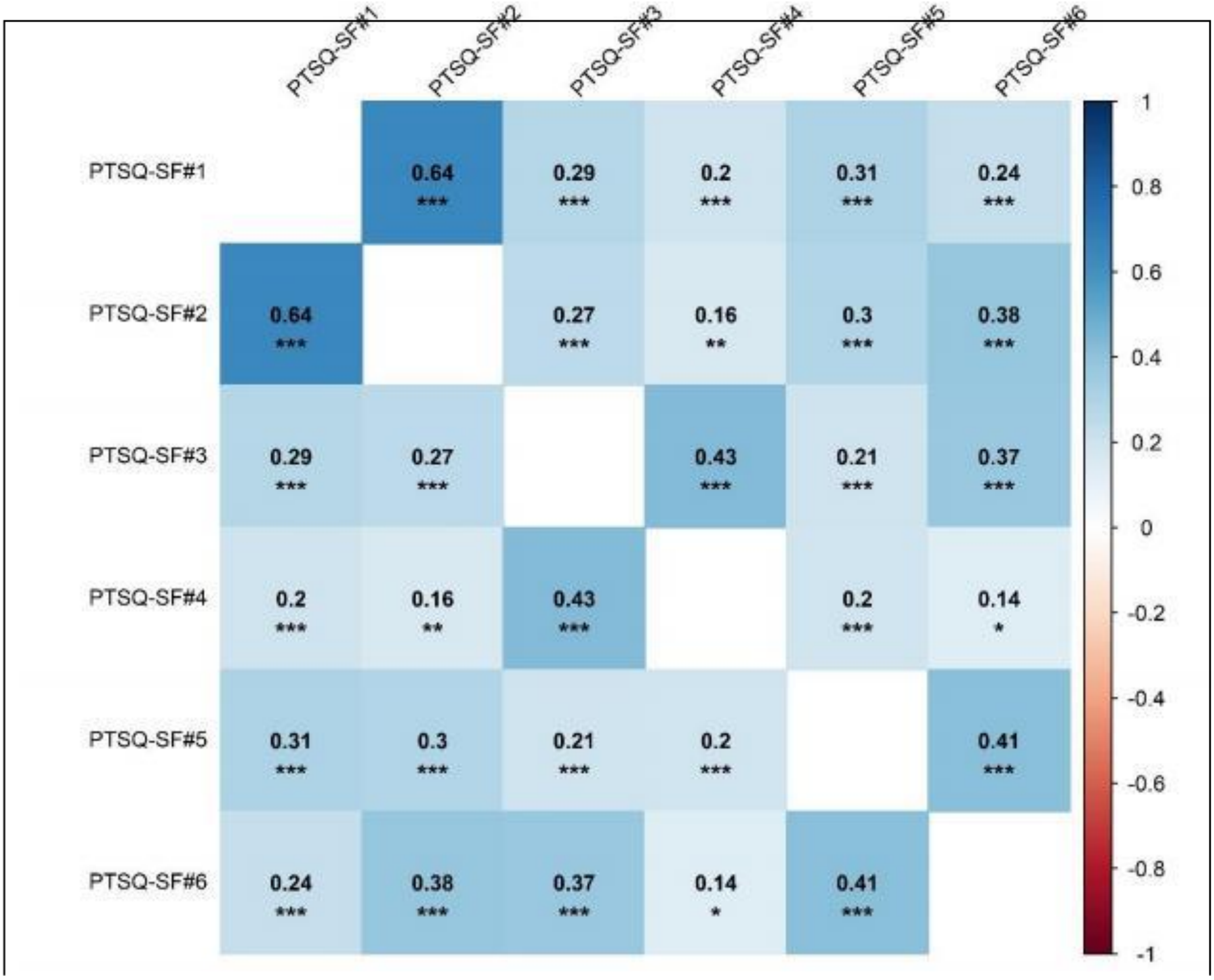

Figure S1. Correlation matrix between items of the PTSQ-SF.

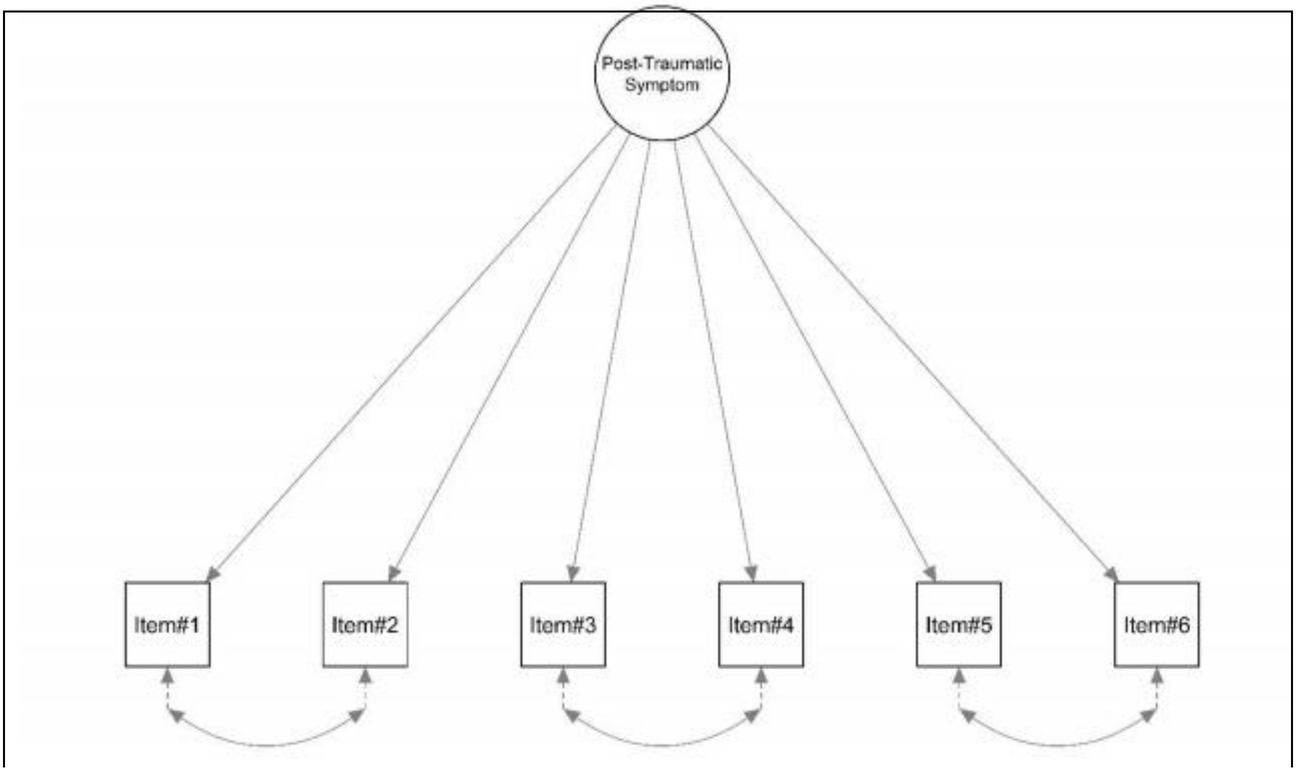

Figure S2. Conceptual representation of the PTSQ-SF

Table S1. Confirmatory factor analysis (CFA) results.

	Descriptives				CFA	
	M	SD	Sk	K	λ	R^2
PTSQ-SF#1	3.57	1.102	-0.327	-0.631	0.540	0.291
PTSQ-SF#2	3.33	1.102	-0.154	-0.637	0.604	0.365
PTSQ-SF#3	2.79	1.214	0.170	-0.817	0.569	0.324
PTSQ-SF#4	2.77	1.393	0.244	-1.205	0.342	0.117
PTSQ-SF#5	2.40	1.221	0.551	-0.603	0.566	0.321
PTSQ-SF#6	2.95	1.231	0.004	-0.885	0.654	0.427

Note: M = mean; Sd = Standard deviation; Sk = Skewness; K = kurtosis; λ = standardized factor loading. R^2 = explained variance. All factor loadings are statistically significant with $p < 0.001$.

post-Traumatic symptom Questionnaire (PTSQ-SF)

INSTRUCTIONS: Listed below are reported problems, issues, emotions and difficulties that often afflict people following traumatic and/or stressful life events. Please read each item carefully and indicate **the intensity you have suffered from these problems during the past week – including today**. We ask you to answer all the questions as accurately as possible. Thankyou.

PART I: (you can mark more than one answer) I experienced:

	During childhood/adolescence:		During adulthood:	
Tragic loss of a beloved one	NO	YES	NO	YES
Severe accidents (car accidents and/or domestic accidents)	NO	YES	NO	YES
Serious illnesses	NO	YES	NO	YES
Physical abuse	NO	YES	NO	YES
Psychological abuse	NO	YES	NO	YES
Emotional abuse within the family	NO	YES	NO	YES
Sexual abuse/harassment	NO	YES	NO	YES
(Became a victim of) bullying	NO	YES	NO	YES
War, persecution, forced migration	NO	YES	NO	YES
Catastrophic natural events (e.g., earthquakes, floods, etc.)	NO	YES	NO	YES
Other (I experienced or witnessed other life events that deeply affected me)	NO	YES	NO	YES

<u>PART II</u>: <u>Thinking about the traumatic/stressful event</u> you experienced.

1	2	3	4	5
Not at all	Alittle bit	Moderately	Quite a bit	Extremely

1	I felt unpleasant emotions to every memory of what happened	1	2	3	4	5
2	Even unintentionally, I thought about what happened	1	2	3	4	5
3	I avoided things that might remind me of what happened	1	2	3	4	5
4	I avoided talking about what happened	1	2	3	4	5
5	I over-reacted	1	2	3	4	5
6	I felt watchful and on-guard	1	2	3	4	5

Post-Traumatic Symptom Questionnaire (PTSQ-SF)

ISTRUZIONI: Nella lista che segue, **sono elencati problemi e disturbi che spesso affliggono le persone** in **seguito ad eventi traumatici e/o stressanti** della vita. Le chiediamo di leggerla attentamente e di cercare diricordare **se ne ha sofferto nella scorsa settimana – oggi compreso – e con quale intensità.** Lapreghiamo dirispondere a tutte le domandenelmodo più precisopossibile.

PARTE I: (può segnare più risposte) hovissuto:

	Durante l'infanzia/adolescenza:		Durante l'età adulta:	
Lutti avvenuti in modo tragico	NO	SÌ	NO	SÌ
Gravi incidenti (in automobile e/o domestici)	NO	SÌ	NO	SÌ
Gravi malattie	NO	SÌ	NO	SÌ
Abusi fisici	NO	SÌ	NO	SÌ
Abusi psicologici	NO	SÌ	NO	SÌ
Abusi emotivi in famiglia	NO	SÌ	NO	SÌ
Abusi/molestie sessuali	NO	SÌ	NO	SÌ
(Subito) bullismo	NO	SÌ	NO	SÌ
Guerra, persecuzione, migrazione forzata	NO	SÌ	NO	SÌ
Eventinaturali catastrofici (e.g., terremoti, inondazioni, ecc.)	NO	SÌ	NO	SÌ
Altro (hovissuto/ho assistito ad altre esperienze divita che mihanno fortemente segnato)	NO	SÌ	NO	SÌ

<u>PARTE II</u>: Pensando all'evento traumatico/stressantevissuto...

1	2	3	4	5
Per nulla	Poco	Abbastanza	Molto	Moltissimo

1	Ho provato emozioni spiacevoli ad ogni ricordo dell’accaduto	1	2	3	4	5
2	Anche senza volerlohopensato all’accaduto	1	2	3	4	5
3	Ho evitato cose che potesseroricordarmi l’accaduto	1	2	3	4	5
4	Ho evitato diparlare dell’accaduto	1	2	3	4	5
5	Ho avutoreazioni eccessive	1	2	3	4	5
6	Mi sono sentito/a guardingo/a evigile	1	2	3	4	5
